# Supplementary material for: Dementia is our “biggest expanding caseload”: Core learning for student speech and language therapists
Source: PLoS One. 2025 Dec 8;20(12):e0327090. doi: 10.1371/journal.pone.0327090 (PMC12685208; doi:10.1371/journal.pone.0327090)
Supplement: S1 File — (DOCX) [file pone.0327090.s001.docx]

S1 – MMAT tool annotated to demonstrate how manuscript follows the guidance

Yes See methods

Yes See discussion

Yes See discussion

Yes See discussion

Yes Yes – FG guidelines referenced

Not relevant to the current study

Yes See participant recruitment

Yes See demographics in results

Yes Survey

Yes 16 of 25 UK universities represented

Yes descriptive states

Yes See RQs

Yes See methods and results

Yes See design

Yes See methods

Yes See results

Yes See discussion

Yes See discussion
