# Supplementary material for: Dementia is our “biggest expanding caseload”: Core learning for student speech and language therapists
Source: PLoS One. 2025 Dec 8;20(12):e0327090. doi: 10.1371/journal.pone.0327090 (PMC12685208; doi:10.1371/journal.pone.0327090)
Supplement: S2 File — (PDF) [file pone.0327090.s002.pdf]

Block 1

What University do you teach at?

Is the speech and language therapy course taught as (tick all that apply):

- ☐ BSc degree
- ☐ MSc degree
- ☐ Apprenticeship

How many hours of teaching is there on dementia (provide an estimate for all courses taught)?

☐ On the BSc degree

☐ [ ] On the MSc degree

☐ [ ] On the Apprenticeship

Who delivers the teaching on dementia to the speech and language therapy students (tick all that apply)?

☐ Lecturer who is a specialist clinical academic SLT in dementia

☐ Guest lecturers who is a specialist clinical SLT in dementia

☐ Lecturer who doesn't specialise in dementia but is an SLT by background

☐ Lecturer who doesn't specialise in dementia nor is an SLT- if so what is their background?

[ ]

☐ If the course is taught by different people on your MSc vs BSc vs Apprenticeship please explain

[ ]

Do students get taught:

	Definitely yes	A small amount (we touch on it)	Probably not	Definitely not
Differential diagnosis criteria	☐	☐	☐	☐
Speech and language disorders in different dementia types	☐	☐	☐	☐

	Definitely yes	A small amount (we touch on it)	Probably not	Definitely not
Assessment approaches and tools	☐	☐	☐	☐
The SLT role in differential diagnosis	☐	☐	☐	☐
Cognitive Stimulation Therapy	☐	☐	☐	☐
Communication partner training	☐	☐	☐	☐
Impairment based therapy approaches for communication	☐	☐	☐	☐
Life story books	☐	☐	☐	☐
Compensatory communication strategies and AAC	☐	☐	☐	☐
Dysphagia management in dementia	☐	☐	☐	☐
Palliative care for dementia	☐	☐	☐	☐
Behaviours that challenge	☐	☐	☐	☐
Person-centred care in dementia	☐	☐	☐	☐
Decision-making and mental capacity in relation to dementia	☐	☐	☐	☐
If there are differences across courses please explain	☐	☐	☐	☐

In relation to types of dementia, do they get taught about:

	Definitely yes	A small amount (we touch on it)	Probably not	Definitely not
Alzheimer's dementia	☐	☐	☐	☐
Vascular dementia	☐	☐	☐	☐
Frontotemporal dementia	☐	☐	☐	☐
Primary Progressive Aphasia	☐	☐	☐	☐
Lewy Body Dementia	☐	☐	☐	☐
Other dementias - please list ______	☐	☐	☐	☐
If there are differences across courses please explain ______	☐	☐	☐	☐

How do you teach students about the SLT role in dementia and who they should work with (e.g. lectures, problem based-learning, lectures from people with lived experience, simulation etc).

______

Do the students get taught about:

	Definitely Yes	A small amount (we touch on it)	Probably not	Definitely Not
The dementia NICE guidance	☐	☐	☐	☐
The RCSLT dementia guidance	☐	☐	☐	☐
The PPA practice principles	☐	☐	☐	☐
If there are differences across courses please explain	☐	☐	☐	☐

Do students attend specialist dementia placements?

- ☐ Yes
- ☐ Some but not all students
- ☐ Don't know
- ☐ No
- ☐ If there are differences across courses please explain

Do you include people with dementia and/or their care partners (partner, spouse, family or friend) in your

lectures?

- ☐ Yes, we have people with dementia (possibly with care partners) in lectures
- ☐ Yes, we have people with dementia (possibly with care partners) on video recordings in lectures
- ☐ Yes, we have care partners ONLY in lectures
- ☐ [ ] Yes, other - please define
- ☐ [ ] No we do not, please explain why
- ☐ If there are differences across courses please explain [ ]

Would you be interested in sharing resources with other lectuers at other universities?

- ○ Yes
- ○ Maybe
- ○ No

Is there anything relevant to the dementia teaching on your SLT courses that you feel is unique or useful to share?

Do any of the students find any aspects of the teaching distressful, if so please explain.

Are there any other local challenges around the teaching on dementia?

Are there any other teaching methods, materials or tasks in relation to dementia, that you would like to comment on with respect to your programmes/courses?
