# Supplementary material for: Dementia is our “biggest expanding caseload”: Core learning for student speech and language therapists
Source: PLoS One. 2025 Dec 8;20(12):e0327090. doi: 10.1371/journal.pone.0327090 (PMC12685208; doi:10.1371/journal.pone.0327090)
Supplement: S3 File — (DOCX) [file pone.0327090.s003.docx]

Supplementary materials 2: Focus Group Topic Guide

Dementia Education Study

Focus Groups Topic Guide

11^th^ and 13^th^ September 2024

Facilitator: RT,

Platform: zoom

Duration: 90 minutes

AV & JK will attend as technical support and/or participants, but the meeting will be led by RT.

Start:

“Hello and welcome to this focus group meeting for the Dementia Education study in speech and language therapy. As you all know, we, are coordinating this study with the aim of developing some consensus on the teaching of dementia on UK SLT courses. We started by getting in contact with all the universities who run speech and language therapy courses and invited you all to participate in the study. We circulated a survey to interested parties and have designed a topic guide based on the answers to those questions. *You should have all completed a consent form when you completed the survey. But we have circulated an additional consent form for this focus group meeting- could you all make sure you have signed and returned that to us.*

Before we continue does anyone have any specific questions.

Just to remind you, this discussion will be video recorded and transcribed for analysis. So, before we do anything else, let’s hit record.

Now, given we ourselves teach on this topic, we wanted to be part of the discussion and so have invited RT to facilitate our discussions- so we can join in. The meeting today will finish promptly at 1330.

So, without further ado let’s hand over to RT.”

1. Welcome I’m RT...(brief intro) okay we are going to start with a round of introductions- **just say your name, which uni you are from** to start with. For the purposes of the tape AV/JK- could you start.... Take it in turns

1. Why is dementia different from aphasia? Or other teaching on communication disorders that we do?

1. How important do you think dementia is on the SLT curriculum and what are the absolute essentials we must teach around this topic?

*Probe: Have we got it right? Should we be doing it differently?*

1. Should we be doing anything to enhance the teaching across our courses in your opinions?

1. In relation to the more experiential side- what do you all see as the challenges of seeing people with dementia on placements for students?

*Probe:* What do the students get from meeting people with dementia on placements or in simulations?

*Probe: Can you explain or describe what you mean by simulation on your courses?*

1. Do you think we should be developing any shared resources and what would they look like?

1. That leads nicely into the final question – Have you found that students become upset during the dementia lectures, and how do you think we should be managing this within the profession?

*Probe- Why do you think this is more upsetting than other caseloads we cover?*

*Probe- Is it specific to dementia?*

*Probe- How should we deal with the ‘difficult’ bits of dementia in our teaching in response to this?*

Finish up 10 mins before end of session to leave time for questions.

AV and JK to finish by explaining next steps- we will transcribe the data and using reflexive thematic analysis we are planning to identify common themes, common challenges and what would be the priorities for the profession (like a list of competencies) and shared resources.

Supplementary file 3: Detailed overview of the teaching content reported by respondents

Table 2:

| Students get taught:  Differential diagnosis  Yes  Small amount    Speech and language disorders in different dementia types  Yes  Small amount    Assessment approaches and tools  Yes  Small amount  The SLT role in differential diagnosis  Yes  Small amount  Probably not    Person Centred Care in dementia  Yes    Dysphagia management  Yes  Small amount | 11  5        14  2      14  2      12  2  2      16      14  2 |
| --- | --- |
