# Supplementary material for: Dementia is our “biggest expanding caseload”: Core learning for student speech and language therapists
Source: PLoS One. 2025 Dec 8;20(12):e0327090. doi: 10.1371/journal.pone.0327090 (PMC12685208; doi:10.1371/journal.pone.0327090)
Supplement: Attachment — Submitted filename: PLOS Review.docx [file pone.0327090.s004.docx]

I thank the editor for giving me this opportunity to review the manuscript titled, “Dementia is our 'biggest expanding caseload': Core learning for student speech and language therapists”. While the topic is of interest and relevant to the current context where SLPs are seeing an exponential increase in the number of dementia patients in their practice, the manuscript has several methodological concerns that need to be addressed. I have first given my general/overall concerns pertaining to the manuscript, followed by section-wise comments. I hope these comments will help improve the quality of the manuscript

Overall Comments

**Study Design:** The Authors have mentioned the study design as “explanatory sequential mixed methods”, which is reasonable, but I don’t see how the quantitative data is explained by the qualitative information here, which is the crux of a mixed methods study. Additionally, I don’t see a clear description of how survey findings were used to inform the topic guide and later develop themes. Describe where and how integration occurred at the analysis or interpretation stage. Map each research question to the phase of the study that answers it

**Inconsistencies in quantitative data reported**: Authors state **two focus groups with 6 and 12 attendees (total 18)**, yet the Results header says, **“Nineteen participants”**. Make these numbers consistent throughout. In the survey, you report 16 universities responded, but in Table 1, the line “Both BSc and MSc” lists 8, whereas earlier text says **5**. Check all these values and report them correctly

**Procedure:**

1) Briefly detail the **Zoom** setup. There is established guidance on conducting rigorous online focus groups that you can cite.
2) Transcription and verification. Indicate transcription approach, de-identification, and accuracy checks. It is mentioned in the appendix that it was done manually; however, it is nowhere mentioned in the manuscript.
3) Your write-up uses RTA, but some phrasing implies a goal to “maximize objectivity” and “triangulate themes” via participant feedback. In RTA, researcher subjectivity is a resource, not a problem to eliminate; “inter-rater reliability” or “theme validation” by participants is not required and can conflict with RTA’s epistemology. If you shared themes with participants, frame this as a stakeholder consultation that informed interpretive refinement, not as verification. Also, avoid presenting multiple coders as a validity check; instead, explain how analytic conversations among AV, RT, MG, and JK deepened interpretation. Update language accordingly. Data Triangulation essentially used data from multiple sources, not multiple assessors. This needs to be revised.

**Potential Bias in data**: V and JK attended both groups mentioned as tech support and were also **participants** in Group 1; this is a **power-dynamics risk** and may shape discourse. Describe how this was managed (e.g., moderator control, ground rules, whether AV/JK refrained from speaking during interviews).

**Results:** The results are exhaustive. Almost, I feel the results can be better represented using tables showing codes, themes, and sub-themes rather than a 12-page summary of the results. Quotations should not overwhelm your narrative. The bulk of your results section should be your interpretation and thematic synthesis, with quotations serving as evidence.

**Section-wise Comments**

Abstract: The research questions can be removed from the abstract, and the authors can briefly mention the aims and objectives of the study. A lot of points in the abstract six themes labelled 1.,2., 3., … and then five principles…1,2,3…

Introduction:

Line 82: The study begins with mentioning the marked increase in low- and middle-income countries. However, this point does not align with the research question. This can be mentioned later as a global concern, and first, the data pertaining to the area of study needs to be highlighted.

Line 102: The font style/ size seems to be different. Kindly cross-check and maintain consistency.

Line 106: Is this expansion into different fields and client groups the author’s perception? If not. Some relevant articles should be cited here to support the claim of this evolution of the profession across different fields.

Line 111: Again, “progressive neurological conditions….. have been a later addition” is a claim that needs to be supported with evidence from the literature

Line 117: Abbreviations appear without proper reference. In line 113, use the abbreviation RCSLT in brackets first. Similarly, NICE is to be expanded here.

Line 132: “It seems logical…” Instead of saying this, the authors should write from relevant literature regarding barriers and discuss briefly how teaching efficient communication can overcome these barriers

Lines 170 to 174: These look like research questions and not aims. Kindly rephrase or mention that the study aimed to answer the following research questions.

Method:

Line 176: Explanatory sequential mixed-methods study: An explanatory sequential mixed-methods design is a reasonable choice (survey → focus groups). However, the manuscript needs to demonstrate integration, not only sequence. Please make explicit how survey results *built* the focus-group guide (name the specific survey items that led to specific probes), and where and how quantitative and qualitative findings were integrated in interpretation

You cite the MMAT; please report how MMAT criteria were applied to judge quality across the two components (not just that you “followed guidance”). Even a brief appendix showing item-by-item judgments will help.

Line 190: An email was circulated to the Committee of Representatives of Education in Speech and Language Therapy:” Are these Email IDs available on the RCSLT website? If so, specify that; otherwise, mention how these email IDs were obtained. Example if obtained from official websites of HEIs or elsewhere

Line 193-194; Some explanation is needed here. What was the duration from sending out the emails and getting the responses? After how many days/ weeks did the authors mail the remaining six HEIs? Were any reminders sent, or did everyone respond to the first email? The timelines need to be specified. Response rate/ completion rate of the survey needs to be explicitly reported

Line 197: 24 from 19 HEIs.. Further distribution is needed to specify bias in the data. What was the distribution of these participants across 19 institutes?

Line 199: First mention that the study had different phases… a brief survey followed by FGDs, to explain the procedure.

Line 202-203: The authorship should be based on ICMJE guidelines. Please specify the roles and responsibilities of each author as per these guidelines. Giving out authorship based on participation is not only unethical but also risks potential bias in the study.

Line 205: “Senior” in terms of age or experience in the dementia related field. Please specify

Line 207-212: The questions are vaguely described here in the method section. Did the questionnaire have any sub-sections? The manuscript lacks information on the development of the survey. How were these questions identified, and who validated the survey questionnaire? Please report instrument development (content mapping to aims), validations, and any revisions or CVI scores.

Line 210: They were also asked to provide a brief comment on the local challenges around dementia teaching and whether they had any relevant resources they felt were unique and useful to share. Where are the findings from these sections reported? I suppose this would constitute qualitative information reported by the participants. How was this taken up for analysis?

Line 218: Who were these speech and language therapy researchers (RTs)?

Line 231: The first FGD was attended by 6 participants, and the second by 12. What happened to one participant (12+6=18 out of 19)?

Line 233: Though authors have reported the bias as being the participants, out of the six participants in the first FGD, 2 were the study authors, which could have significantly biased the obtained data in the first round. Hence, I am skeptical about how the conversations were steered in this FGD. Describe clearly concrete reflexive safeguards (e.g., did AV and JK refrain from speaking?). Also, the description of the participants and their representativeness from HEIs is lacking.

Line 244: Kindly specify how the transcription was done. Though it is mentioned briefly in the appendix authors should clearly describe the method here. Why was no software like Atlas TI used?

Line 245: Though the use of RTA is apt, the authors should

Analysis: The Data Triangulation Process looks more like reliability. Again, this is mentioned in the appendix; however, a description of how it was done is warranted in the methodology section

Results:

Line 261: Further distribution of how the 19 participants were distributed across the 16 universities needs to be specified.

Table 1: Incorrect descriptive statistics reported. Means are reported with a range. What is the meaning of “specialist”? As the readers may be from different backgrounds, the authors need to specify what they mean by this.

Line 271: Refer to the comment given for line 231

Line 272: Authors need to specify how these themes were identified. A general process in qualitative research involves the generation of initial codes, which are further divided into themes. The codes are nowhere mentioned in the manuscript (except in the appendix), so as a reader, I am unable to understand how these themes emerged.

Line 282: The following provides a detailed description of each theme, accompanied by relevant quotations from participants (using the letters A-S as identifiers): All paerici

The result section is approximately 24 pages. exhaustive and is not summarised in a way that qualitative research should be reported. Authors should identify

Discussion:

The authors can integrate the findings from the mixed method design in this section.

Line 783: The prevalence data reported is repetitive and already mentioned in the introduction. Kindly rephrase and avoid repetitiveness.

Line 836: Five core components or ‘pillars’ were synthesized from participants’ focus-group discussions. How was this synthesis done? There is no mention of such themes or codes in the analysis process. How did authors come up with this during the writing process of the manuscript?

Limitations of the study to be discussed in detail

Table and figures

Tables and figures need to be significantly revised. Table 2 is not referenced anywhere in the manuscript.

Appendices

Appendices should support information from the manuscript. Currently, the important information that is significant in understanding the analysis and interpretation of findings is missing from the main body of the manuscript and added in appendices.
